# Female fertile phase synchrony, and male mating and reproductive skew, in the crested macaque

**DOI:** 10.1038/s41598-021-81163-1

**Published:** 2021-02-19

**Authors:** James P. Higham, Michael Heistermann, Muhammad Agil, Dyah Perwitasari-Farajallah, Anja Widdig, Antje Engelhardt

**Affiliations:** 1 Jr Research Group on Sexual Selection, Reproductive Biology Unit, German Primate Centre, Göttingen, Germany; 2 Department of Anthropology, New York University, 25 Waverly Place, New York, NY 10003 USA; 3 Endocrinology Laboratory, German Primate Centre, Göttingen, Germany; 4 Faculty of Veterinary Medicine, Bogor Agricultural University, Bogor, Indonesia; 5 Primate Research Center, Bogor Agricultural University, Bogor, Indonesia; 6 Faculty of Mathematics and Natural Sciences, Bogor Agricultural University, Bogor, Indonesia; 7 Research Group Primate Behavioural Ecology, Department of Human Behaviour, Ecology and Culture, Max Planck Institute for Evolutionary Anthropology, Leipzig, Germany; 8 Research Group of Behavioural Ecology, Institute of Biology, University of Leipzig, Leipzig, Germany; 9 School of Natural Sciences and Psychology, Liverpool John Moores University, Liverpool, UK

**Keywords:** Animal behaviour, Behavioural ecology, Anthropology, Sexual selection

## Abstract

High social status is the primary determinant of reproductive success among group-living male mammals. Primates living in multimale–multifemale groups show the greatest variation in the strength of this link, with marked variation in reproductive skew by male dominance among species, dependent on the degree of female fertile phase synchrony, and the number of competing males. Here, we present data on two groups of wild crested macaques (*Macaca nigra*), living in the Tangkoko Reserve, Sulawesi, Indonesia. We investigated male monopolization of fertile females in 31 cycles of 19 females, and genetic paternity of 14 offspring conceived during the study period. We show that female fertile phase synchrony was low, that females had few mating partners in their fertile phase, and that dominant males monopolized a high proportion of consortships and matings, resulting in marked and steep mating and reproductive skew. We conclude that female cycle asynchrony provides the opportunity for strong direct male–male competition in crested macaques, resulting in monopolization of females by dominant males, consistent with their marked sexual dimorphism. Our study provides a test of the underlying factors that determine the relative occurrence and strength of different mechanisms of sexual selection, and the phenotypes that evolve as a result.

## Introduction

Sexual selection theory predicts conflict between members of one sex for reproductive opportunities with the other. For many mammals, this often manifests as competition between males for females, which represent the limiting resource in reproduction^1^. For group-living mammals, social status usually determines access to females during receptive periods, with males of high social status obtaining most sires, a phenomenon often known as reproductive skew (e.g. kangaroos^2^; coatis^3^; feral cats^4^). The degree to which males and females associate, and the extent to which males can monopolize females, are key determinants of the extent of dominant male reproductive success^5^. Given that males may only be able to monopolize one female at a time, it has been suggested that in species living in multi-male multi-female groups with any overlap between females in periods of fertility, males ranked higher in the dominance hierarchy gain access to females before lower ranked males in a sequential Priority of Access (PoA) system^6^.

Non-human primates are a particularly interesting group in which to study the related issues of female cycle synchrony and male reproductive skew, as primate species show an unusually high level of variation in such skew^7,8^. This variation can be attributed to the fact that primate species vary a great deal in the number of females present in groups and mating seasonality (and hence fertile phase synchrony)^9,10^, as well as in the number of males found in social groups (and hence the number of competitors for a dominant male)^7^. This variation is ultimately caused by ecological factors, with seasonality of breeding being determined by seasonality of food resources^11^ and number of females (itself determined by within and between group competition^12^) determining the number of males^13,14^. When females are highly asynchronous in the timing of their fertile phases, high-ranking males may be able to monopolize these periods, leading to marked reproductive skew. In contrast, when females are highly synchronous in the timing of their fertile phases, monopolization may be difficult, leading to lower reproductive skew^8^.

A key component of male monopolization in primates is in the form of consortships, in which high-ranking males associate with fertile females, and travel, feed, and rest with them, preventing them from mating with other males^15^. Females are likely to have their own preferred order of mating partners, which may be based on genetic or other quality, or compatibility^16^. Female mate choice preferences may concur with the order of the male dominance hierarchy when high-ranking males are of high quality and are highly competitive, but may not when dominance rank is not a good proxy of male quality (e.g. Assamese macaques^17^). A key question in determining whether female preferences align with the dominance hierarchy and the outcome of PoA, surrounds the maintenance of consortships, and whether these are being maintained by males, females, or both.

While the degree of cycle synchrony may be the starting point for selection for different male strategies, and hence the degree of sexual dimorphism, this may then create a feedback loop onto female strategies. The ability of females to exert more *direct* choice over their mating partners may then start to depend on this degree of sexual dimorphism, with increasing monopolizable potential in those species where dimorphism is high^18^. Consistent with this, those species with high levels of sexual dimorphism tend to have the highest levels of reproductive skew (e.g. mandrills^19,20^). However, this may not always be the case (see^10^). One factor that may influence the relationship between sexual dimorphism and reproductive skew is female ovulatory signalling. Females may signal ovulation clearly, probabilistically^21^, or with less precision, and so through the use of signals may determine the extent to which males are able to recognize and hence monopolize fertile periods. The overall degree of monopolization experienced by females may therefore represent the outcome of mechanisms of male–male competition, combined with the outcome of mechanisms of both direct and indirect female mate choice. Key measures of this degree of monopolization include the number of different partners that a female mates with during the cycle, (and especially during fertile phases), the degree of mating skew by male dominance rank, and ultimately, reproductive skew.

Among the macaques (*Macaca *spp.), males of high social status generally have higher reproductive output^22^, but there is considerable variation in the percentage of sires obtained by alpha males between species (e.g. 6–25% in *M. sylvanus*^23–25^; 20–30% in *M. mulatta*^26–28^; 60–90% in *M. fascicularis*^29,30^). Correspondingly, there is high variation in male sexual dimorphism, with males of some species similar in size and weaponry to females, while males in other species are more than twice as large as females and have much bigger canines^31,32^. Though there have been studies of these variables in several macaque species, the full extent of variation and its causes remains poorly understood. The Sulawesi macaques are an important study taxon in this regard as they represent almost a third of extant macaque species^33^, and differ from other macaques in exhibiting more marked sexual dimorphism, while also exhibiting highly socially tolerant female–female relationships^34,35^. Their reproductive biology is largely unknown.

Here, we assess the level of female cycle synchrony, the extent to which males, especially the alpha male, are able to monopolize female fertile periods, and the extent of male mating and reproductive skew, in a wild population of one species of Sulawesi macaques, the crested macaque (*M. nigra*). The crested macaque has been historically subject to fewer studies than many other macaque species. Many of the more commonly-studied species, such as rhesus, Japanese, and Barbary macaques, are all seasonal breeders with a relative low level of sexual dimorphism^36^. In contrast, crested macaques and is a non-seasonally breeding species with a high degree of sexual dimorphism. Males attain full body size in their natal groups, then disperse to compete aggressively and directly for alpha-male status, with all observed alpha-male replacements coming from outside the group^37^. Incoming males are infanticidal, with alpha-male replacement the number one cause of infant mortality^38^. Consistent with the importance of male dominance rank, males give clear signals of dominance such as loud calls^39^, and males and females both defend groups in inter-group encounters^40^. Females are known to exhibit multiple signals of fertility, including very large sexual swellings, copulation calls, and numerous proceptive behaviors, which covary with the timing of the fertile phase with a high degree of reliability^41^. As such, females accurately signal their intra-cycle conceptive probability to males. Females exhibit an average 2.4 (range 1–4) cycles before conception following lactational amenorrhea^42^. We studied two groups of crested macaques between 2006 and 2007 in the Tangkoko Reserve, North Sulawesi, Indonesia.

Our study was structured around the following aim and predictions: (1) to assess the degree of *female ovarian cycle synchrony*, as measured by overlap of maximal swelling periods. We predicted synchrony to be low, based on preliminary observations of year-round births^42^; (2) to determine patterns of *consortships* across different phases of the female cycle. Consistent with low female synchrony and high monopolization potential, we predicted that females would be consorted by dominant males for the majority of their fertile phases; (3) to determine whether males or females are responsible for the *maintenance of consortships*. Since dominance acquisition is highly physically-competitive in male crested macaques^37^, we assume that high-ranking males are likely to be of high quality. On this basis, we predicted that such males would be the preferred partners of females, such that both males and females would maintain consortships; (4) to assess the *number of mating partners* across the cycle. Since we predict high male monopolization, we predict that there will be few mating partners and that these will be high ranking males, especially during fertile phases; (5) to assess *mating skew*, and to compare it to that predicted by the PoA model. We predicted that mating skew would be steep, and that it would be higher than that predicted by the PoA model, due to female preferences for high-ranking males further steepening already steep skew; (6) finally to assess *reproductive skew*. Consistent with steep mating skew, we similarly predicted steep reproductive skew.

## Results

### Female ovarian cycle synchrony

**Figure 1:**
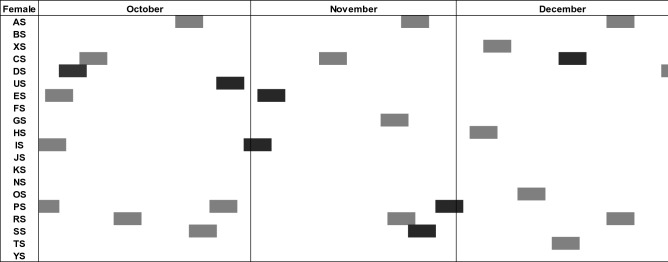
Overlap of fertile phases in group Rambo I during October to December 2006. Grey boxes indicate fertile phases of non-conceptive cycles and black boxes indicate those of conceptive cycles. In both groups, a maximum of three females were in their fertile phase on any given day of the study period.

Across both groups, on any one day there was an average of 16.8 ± 0.0 (mean ± STD; range 14–20) females present during observation at any one time; 3.3 ± 0.1 (range 0–9) females exhibiting a swelling; 1.3 ± 0.1 (range 0–5) exhibiting maximal swelling; and 0.5 ± 0.0 (range 0–3) females being in the fertile phase of their cycle. (See Fig. 1 for an exemplary three months in group Rambo I). Only 0.2 ± 0.4 (range 0–2) females were in the fertile phase of their conception cycle on any one day and only during 3.9% of days in which a female was in the fertile phases of her conceptive cycle was there a second female in the same cycle stage and type. The mean operational sex ratio was 0.06 ± 0.00 (range 0.00–0.33).

### Consortships

Females consorted with males a mean 83.5 ± 3.3% of time during the four day fertile phase, compared with 60.1 ± 6.2% of time in the five days preceding the fertile phase, and 28.6 ± 4.4% of time in the five days following the fertile phase. Separating cycles according to whether they were conceptive or not shows that for conceptive cycles these figures were 87.1 ± 4.1% (fertile phase), 58.8 ± 8.7% (pre-fertile phase), and 32.2 ± 7.0% (post-fertile phase), and for non-conceptive cycles were 79.8 ± 5.2% (fertile phase), 61.5 ± 9.3% (pre-fertile phase), and 24.3 ± 6.5% (post-fertile phase). A Linear Mixed Model with zero-inflated Poisson error structure showed that male likelihood of consorting females varied according to cycle phase (β = 1.7231, lower CI = 1.2824, upper CI = 2.1562, p < 0.001), as did the time they spent consorting females (β = − 0.4699, lower CI = − 0.6344, upper CI = − 0.2984, p < 0.001), such that males were more likely to consort in the fertile phase, and consorted females for longer when consorting. However, there were no differences in consort time across all males between conceptive and non-conceptive cycles, both for the zero-inflated (β = − 0.1273, lower CI = − 0.9359, upper CI = 0.6720, p = 0.753) and Poisson (β = − 0.2361, lower CI = − 0.5672, upper CI = 0.0769, p = 0.148) parts of the model.

**Figure 2:**
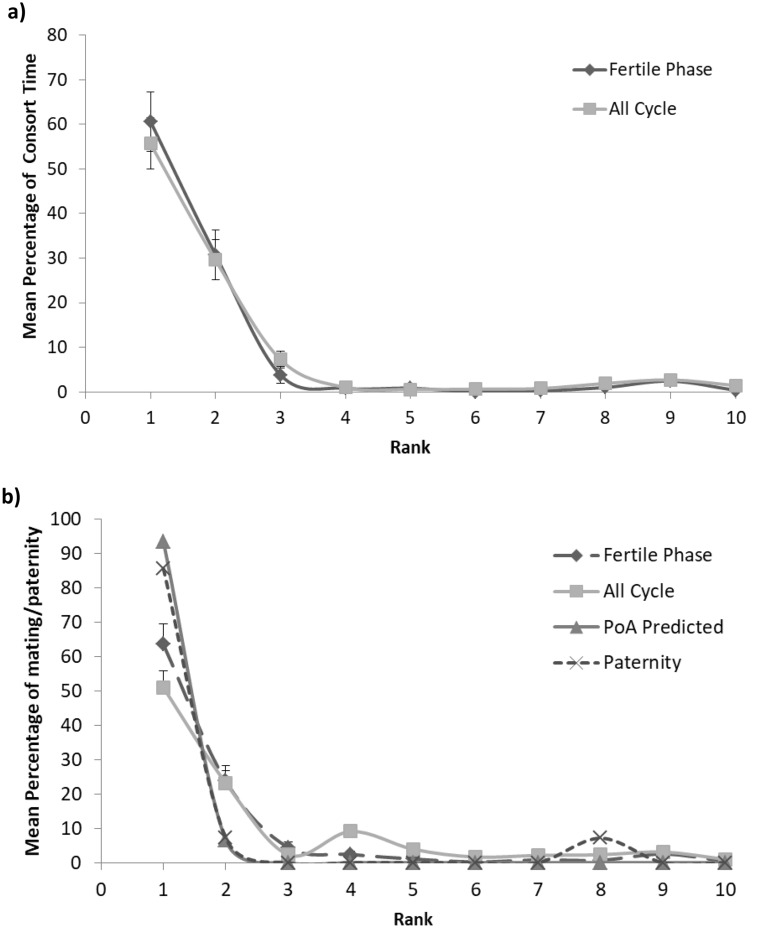
Percentage of consort time (**a**) and matings and paternity (**b**) obtained by males of each rank for the whole 14 day period, and the 4 day fertile period, separately (mean and standard deviation). The mating and paternity plot (**b**) also includes the percentage predicted under the PoA model and the percentage of infants sired.

**Figure 3:**
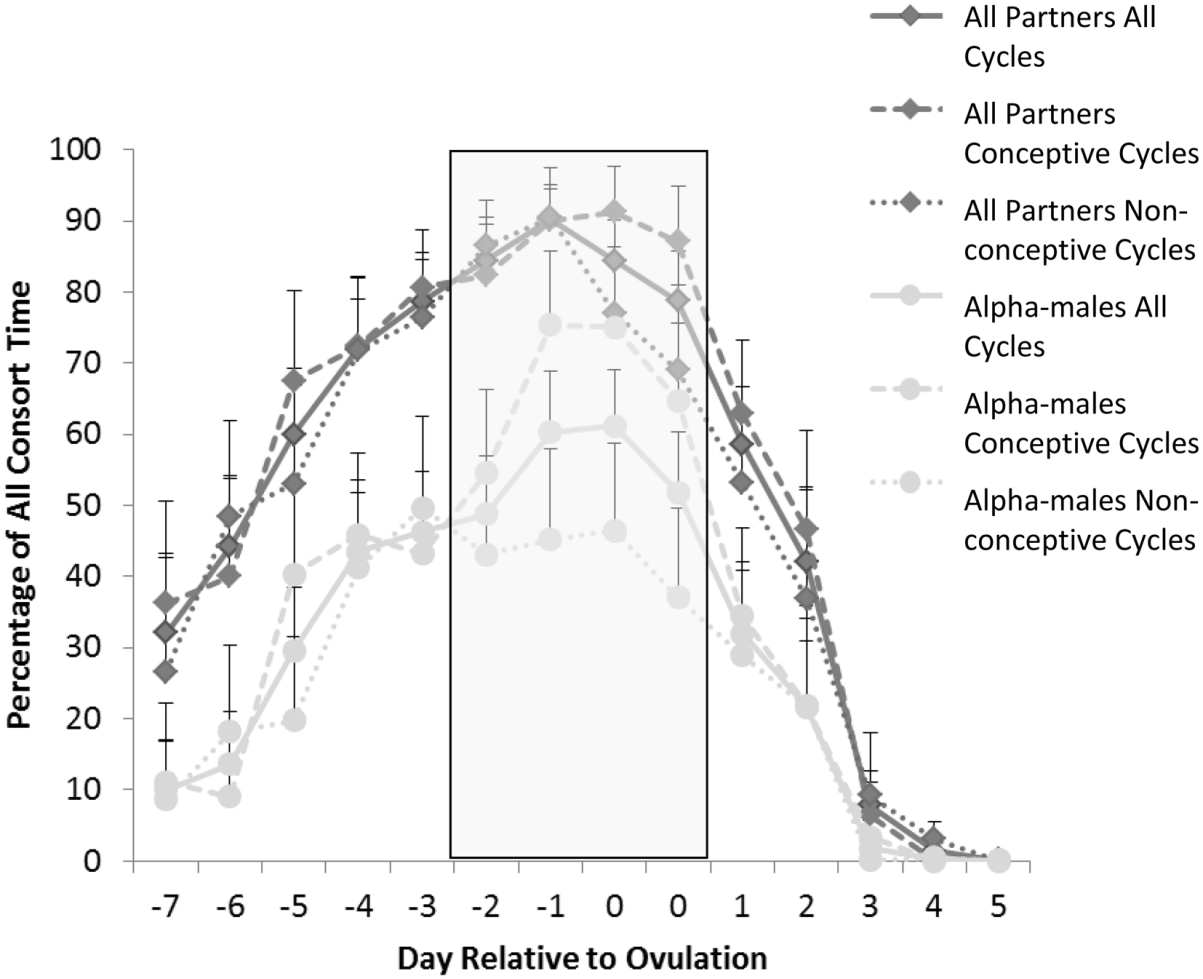
Percentage of time females spent in consort with all consort partners and with alpha-males in all cycles, as well as in conceptive and non-conceptive cycles separately, for each day with respect to the 2 day ovulation window (mean and standard deviation). The grey box indicates a presumed 4 day fertile period.

Consorts were strongly skewed by rank both across the whole 14 day period surrounding ovulation, and within the four day fertile phase (top six individuals only; all 14 days, r = − 0.943, p = 0.005; fertile phase r = − 0.943, p = 0.005; Fig. 2a). Alpha-males obtained a mean of 55.7 ± 5.8% of all consort time across the whole 14 day period around ovulation, and a mean of 62.0 ± 6.7% of consort time during the fertile period. Separating cycles according to whether they were conceptive shows that alpha-males obtained 48.1 ± 10.4% of consort time during the fertile period in non-conceptive cycles and 71.6 ± 8.5% of consort time during the fertile period in conceptive cycles (Fig. 3). A Linear Mixed Model with zero-inflated Poisson error structure showed that for alpha-males, the likelihood of consorting females varied according to cycle phase (β = 1.4713, lower CI = 1.0685, upper CI = 1.8724, p < 0.001), as did the time they then spent consorting females (β = − 0.3845, lower CI− 0.6651, upper CI = − 0.0944, p = 0.008), such that alpha-males spent more time consorting females and were more likely to consort in the fertile phase. Unlike for all males, though, alpha-males were in general not more likely to consort females in conceptive than non-conceptive cycles (β = − 0.1041, lower CI = − 0.8661, upper CI = 0.6277, p = 0.782). However, when they did consort with females, they consorted females for longer in conceptive than non-conceptive cycles (β = 0.9630, lower CI = − 1.4611, upper CI = − 0.4723, p < 0.001; Fig. 3).

### Maintenance of consortships

Both males and females maintained consortships, though the majority of consort time was maintained by males. Males maintained consortships 83.6 ± 2.3% of the time during the fertile phase, 78.4 ± 3.8% of the time during the pre-fertile phase, and 64.6 ± 7.7% of the time during the post-fertile phase. As such, females increased their own maintenance of consortships when they were not fertile. Separating these figures for conceptive and non-conceptive cycles shows that in conceptive cycles, males maintained consortships for 86.1 ± 3.1% (fertile phase), 76.5 ± 6.4% (pre-fertile phase) and 65.1 ± 9.3% (post-fertile phase) of the time, while in non-conceptive cycles, males maintained consortships for 80.6 ± 3.4% (fertile phase), 80.5 ± 4.2% (pre-fertile phase) and 63.3 ± 11.4% (post-fertile phase) of the time. A Linear Mixed Model with zero-inflated Poisson error structure showed that males who were consorting invested with longer periods of time maintaining those consorts (β = − 0.3572, lower CI = − 0.5249, upper CI = − 0.2058, p < 0.001) and were more likely to maintain consorts according to cycle phase (β = 1.8842, lower CI = 1.4101, upper CI = 2.3960, p < 0.001), with a higher degree of consort maintenance in the fertile phase. However, there were no differences in male consort maintenance between conceptive and non-conceptive cycles, both for the zero-inflated (β = 0.1258, lower CI = − 0.6412, upper CI = 0.8626, p = 0.766) and Poisson (β = 0.0462, lower CI = − 0.2497, upper CI = 0.34730, p = 0.744) parts of the model.

### Numbers of mating partners across the cycle

Females exhibited a mean of 3.5 ± 0.4 (range 1–10) mating partners across the whole fertile phase, with a mean 2.0 ± 0.1 (range 1–6) mating partners per each day of the fertile phase. This compares with a mean 5.4 ± 0.5 (range 0–9) mating partners for the five days preceding the fertile phase (2.8 ± 0.2, range 0–7), and 5.5 ± 0.6 (range 0–10) mating partners for the five days following the fertile phase (3.4 ± 0.4, range 0–10). Separating cycles into cycle type (conceptive vs non-conceptive) shows that females had a mean 3.1 ± 0.5 (fertile phase), 5.8 ± 0.7 (pre-fertile phase) and 5.8 ± 0.8 (post-fertile phase) partners in conceptive cycles and 3.9 ± 0.4 (fertile phase), 5.0 ± 0.5 (pre-fertile phase) and 5.3 ± 0.6 (post-fertile phase) partners in non-conceptive cycles. Repeated-measures ANOVA showed that the total number of partners females had in each cycle phase was significantly different (F = 6.15, df = 2, p = 0.004) but did not reveal a significant effect of cycle type (F = 0.92, df = 2, p = 0.405). Paired t-tests showed that the total number of partners was lower in the fertile phase than either the pre-fertile (t_30_ = − 3.04, p = 0.005) or post-fertile phase (t_30_ = − 2.71, p = 0.011), whereas the pre- and post-fertile phases did not differ in this respect (t_30_ = − 0.25, p = 0.807).

### Mating skew

During the study period we observed 4976 matings during the 14 day period around ovulation, including 2315 matings during the four day fertile period. Almost all matings were from in-group males, with almost no out-group mating. Matings were highly skewed by rank (all 14 days, r = − 0.829, p = 0.042; fertile phase, r = − 1.0, p < 0.001; Fig. 2b). The alpha-male obtained a mean 51.0 ± 5.0% of matings during the whole 14 day period around ovulation, and a mean 63.5 ± 6.0% of matings during the fertile period. Separating cycles according to whether they were conceptive or not shows that alpha-males obtained 56.3 ± 8.8% of matings during the fertile period in non-conceptive cycles and 70.3 ± 8.1% of matings during the fertile period in conceptive cycles. A Linear Mixed Model with zero-inflated Poisson error structure showed that alpha-males were not more or less likely to mate according to cycle phase (β = − 0.4668, lower CI = − 1.1910, upper CI = 0.2160, p = 0.167), but mated at higher frequencies in the fertile phase (β = − 0.5278, lower CI = − 0.6777, upper CI = − 0.3826 p < 0.001). Alpha-males were not more likely to mate in conceptive than non-conceptive cycles (β = 0.6884, lower CI = − 0.7467, upper CI = 2.1814, p = 0.331), and when they did mate, did not mate at higher rates in conceptive cycles (β = − 0.0267, lower CI = − 0.3496, upper CI = 0.3284, p = 0.879). Alpha-males mated less often during the fertile phase (63.5%, see above) than would be predicted (89.8%) from the Priority of Access model based on patterns of female fertile phase overlaps. Similarly, the steepness of observed fertile phase mating skew (r = − 0.733, p = 0.016, see above) was lower than predicted by the Priority of Access model (r = − 0.813, p = 0.004).

### Reproductive skew

Male reproductive success was highly skewed towards alpha-males (Table 1). Of the 14 infants conceived during the study period, 12 (86%) were sired by the group’s alpha-male. The first infant of a nulliparous female was sired by the group’s beta male and a third, the last infant of a very old female, by a low ranking male that had just newly immigrated into the group. Nonacs’ B-index for paternity was high and significantly different from a random distribution (B = 0.508, p < 0.001).

**Table 1:** Results of the genetic paternity analysis for 14 mother–infant pairs.

Mother–infant pair	Group	Sire	Sire rank	Number of loci compared	Number of infant–father mismatches	Paternity likelihood (%)
ES-1	R1	NJ	8	12	0	> 95
GS-1	R1	IJ	1	12	1	> 95
HS-1	R1	IJ	1	12	0	> 95
NS-2	R1	IJ	1	10	1	> 95
RS-1	R1	IJ	1	12	1	> 95
US-2	R1	IJ	1	12	0	> 95
YS-1	R1	IJ	1	12	0	> 95
HD-1	R2	SJ	1	11	0	> 95
ID-2	R2	SJ	1	11	0	> 95
JD-1	R2	BJ	2	11	0	> 95
KD-1	R2	SJ	1	11	0	> 95
MD-1	R2	SJ	1	11	0	> 95
UD-1	R2	SJ	1	11	0	> 95
YD-1	R2	SJ	1	11	1	> 95

The table shows the identity of the mother-infant pair, the mother-infant pair’s group, the most likely father, his rank at the time of conception, and the paternity likelihood as calculated with Cervus 3.0.

## Discussion

Our data show that, as predicted, female crested macaques exhibit low fertile phase synchrony, and probably as a direct consequence of this, are heavily monopolized by high-ranking males. They spend 70% of all time during fertile periods of conceptive cycles in consort with the alpha male, and mate with few partners. As a consequence, and consistent with our predictions, mating, and reproductive skew are very high.

The factors influencing female fertile phase synchrony in nonhuman primate populations are number of females (often correlated with group size in species living in mulitmale multifemale groups), combined with the degree of breeding seasonality^7,8,10^, with breeding seasonality in turn often thought to depend on seasonality in the environmental. This population of crested macaques lives in an aseasonal environment (Engelhardt et al. unpublished data), which may have a causal role in influencing the lack of breeding seasonality, and creating relatively low fertile phase synchrony. With few females being able to conceive at any one time, and the presence of clear signals of the fertile phase^41^, it is no surprise that female fertile periods can be monopolized by the top ranked males. As a consequence, females undergo reduced partner numbers (lower promiscuity) when they are fertile than in other periods of the cycle, and spend a high degree of their fertile phases in consorts that are especially maintained by males. Given that females signal their fertile phase to males through the presence of sexual swellings and proceptive behaviours^41^, this may indicate that females have been selected to further enable their own monopolization by high ranking males. Consistent with the above, consort and mating skew are marked, and alpha males particularly are able to dominate consort and mating activities during fertile periods, and therefore sire the majority of infants. The combination of strong reproductive skew with high levels of sexual dimorphism^31,32^ is consistent with the fact that males usually immigrate into groups from outside and engage in direct contest competition for alpha-male status^37,43^. As such, dominance may be a proxy for male competitiveness and/or quality in crested macaques, such that female preferences for males may coincide with male rank.

Females nonetheless had more than one partner during the fertile period despite theoretical preference for high ranking males among females, and despite high monopolization potential for alpha males. Although it remains unclear how females escape male monopolisation, they may have a selective advantage in obtaining multiple mates even when they have a preferred partner. This may be because it ensures fertilization^21^, induces sperm competition^44^, ensures fertilization by genetically compatible individuals^45^, keeps lower ranking males in the group, who might aid with group defence^40,46^, or reduces the risk of infanticide^21^ Infanticide is known to be the number one cause of infant mortality in this study population^38^.

Our data show that mating skew was less steep, and the proportion of matings obtained by the alpha male fewer, than would be predicted from the PoA model^6^. This model has been tested in a number of non-seasonally breeding primate species where female synchrony is low, and has found broad support among such species (e.g. yellow baboons^47^; mandrills^19^; chimpanzees^48^, though see^28^ for seasonal breeders). Numerous possibilities could explain the lower than expected skew in the present study. One explanation may be our choice of using the fertile periods of females only to calculate the degree of female cycle synchrony for our assessment of the PoA model. The timing of the fertile period is the most biologically important variable for males, but using it to calculate female reproductive overlap inevitably leads to relatively low levels of synchrony compared to assessments so far made on the basis of, for example, observations of all swellings of any size, or of mating in general (e.g.^8,14,21^). Such a high degree of mating monopolization may also be unfeasible due to biological constraints (e.g. the energetic costs of consort strategies and mating^49^), or males may choose not to mate with some females of low priority, (e.g. low-ranking females^30^). Nonetheless, alpha-male paternity was higher and reproductive skew steeper than observed in most other primates living in polygynandrous mating systems (see^10^ Suppl. Mat. for alpha-male paternities; see^20,27,50,51^ for B-indices). Alpha-male paternity was especially high given the number of males present in the groups, a parameter so far thought to be one of the most important determinants of alpha-male reproductive success^8,10^. Crested macaque alpha-males thus seem to be able to control reproduction even in the presence of a large number of competitors.

The consort data show that alpha males (unlike all males, see Fig. 2) consorted for longer periods in conceptive than in non-conceptive cycle. Our data are consistent with results of some other species, including captive olive baboon males, which appeared more attracted to conceptive than non-conceptive cycles^52^, and wild male chacma baboons who consorted more with females during conceptive cycles^53^. Other analyses of our dataset show that, across all males, there is no difference between mating rates between conceptive and non-conceptive cycles^41^. Given the strong mating skew and limited reproductive opportunities for lower ranked males this could be because all opportunities for mating must be taken by lower ranked males. Another possibility is that due to their domination of consort periods during the fertile phase, alpha males have increased information about female cycle status not available to all males. We have previously suggested that consorting males may have access to signals from close range (e.g. olfactory signals) not available to all males that enable them to refine their estimates of female fertility^54,55^. For example only group resident male chacma baboons consorted more with females during conceptive cycles^53^. Relatively clear signals of the likelihood of conception may thus support experienced, dominant males in making decisions on which female to monopolise at what time, and in this way further increase the advantages of high rank for males.

Our data also provide further evidence for a relationship between social styles and reproductive skew, with a highly despotic male-male competitive regime observed in a socially-tolerant species^56,57^. Further theoretical and empirical work should focus on whether these links may be a product of higher relatedness between individuals in species of high reproductive skew (as suggested by^56,57^), or whether the correlation may be a product of both systems deriving from related ecological factors, with food resource seasonality influencing both female-female competition and hence social style, and female reproductive synchrony and hence male reproductive skew.

## Methods

### Data

Data are available and citable from Dryad^58^.

### Study site and population

The study was undertaken as part of the *Macaca nigra* Project at the Tangkoko Reserve at the north-eastern tip of Sulawesi (1° 34′ N, 125° 14′ E). The reserve was established in 1980, comprises an area of 8867 hectares, with a sea boundary of 12 km, and ranges from sea level to an elevation of 1350 m^59,60^. All research was undertaken between July 2006 and July 2007 on two study groups (Rambo I, Rambo II), which were studied previously by other researchers^61,62^. We re-habituated these groups from April 2006 to July 2006, and identified and named all adult group members according to individual characteristics such as size, gait, cuts, missing digits, scars etc. The home range of both groups overlapped and included primary forest, secondary forest and, for Rambo II, also gardens near the village. During the study period, both groups ranged in size from 65 to 70 individuals, with Rambo I consisting of 10 adult males and 21 adult females, and Rambo II 7 adult males and 15 females, the remaining individuals in both groups being subadults, juveniles and infants.

### Male dominance rank

Male dominance ranks varied at times during the study period, including one alpha-male takeover over of one of the groups (R2). Following elsewhere^63^, during each period and for each group separately, we used Elo-ratings to create dyadic dominance interaction. We included agonistic interactions with unambiguous winner and loser and displacement interactions for all adult males present during the given study period. Similar to elsewhere^63^, we included only unidirectional interactions, and did not include conflicts in which there was counteraggression by the loser. Elo ratings were extracted on a given day for analysis, and then males were ranked in order, with those rankings used for analysis.

### Female ovarian cycle synchrony

On each day, swelling status of all females was recorded in both groups. The first day of visible swelling of sex skin was noted by a single observer in close proximity and under excellent observation conditions; maximum swelling size and the beginning of detumescence was also assessed by a single member of the field team. Swellings were categorized as maximal when the area around the anus clearly protruded the rest of the swelling from a side view and when the skin was tight over the entire swelling area. The beginning of detumescence was recognized through wrinkles (re-)appearing on the sex skin followed by rapid deflation of the sex skin tissue.

### Behavioural data collection

We collected behavioural data during 31 ovarian cycles (N = 19 adult females), of which 16 were conceptive and 15 were non-conceptive. We classified cycles as conceptive (N = 16) when an infant was born around 6 months later (see^64^), or when it was the last female cycle (either assessed hormonally, see below, or by the occurrence of anogenital swellings, as *M. nigra* does not exhibit post-conception swellings, see^64^; Engelhardt pers. obs.), or when miscarriage was subsequently observed (assessed by female bleeding from the vagina, N = 3). In cases where a cycle was classified as conceptive based on the birth of an infant, the lack of any further cycles with swelling after that conceptive cycle gives us a high degree of confidence that this was indeed the conceptive cycle. Cycles were classified as non-conceptive (N = 15) when they were immediately followed by another cycle (assessed hormonally or by the occurrence of swellings).

Our sampling covered 417 observation days and 2443 h of behavioural data. Data collection was carried out using handheld data recorders (Psion Workabout Pro M, Psion Teklogix) and using focal animal sampling^65^. We attempted to follow focal females every observational day from dawn until dusk, with actual observed time averaging 5.9 ± 0.1 h per observation day and female. We here focus on the two most important sexual behaviours—consortship^15^ and mating. Consortship was defined here as a sexually active female and adult male staying in proximity up to 10 m for at least 10 consecutive minutes and aligning their directions of forward movements (i.e. grooming after mating by one or both mating partners was not categorized as consortship if the two individuals did not move on into the same direction afterwards; see also^41^). Data on consortship were collected using instantaneous sampling^65^ with an interval of one minute and mating was recorded by all occurrence sampling during focal observations. In the case that during a consortship, the female climbed a tree and was not followed by the male, but still closely watched by him, the consortship was noted as continued if there was no other male closer to the female than her partner and if the two individuals continued to stay in proximity once the female returned to the ground. In the case that more than one male stayed in proximity to the female and aligned the direction of movements with her, the highest ranking of the males present was recorded as the main consort partner. In these cases, the lower ranked male was often trailing in the wake of the consort pair, and might be considered to be undertaking the role of a 'follower male'^66^. In order to investigate the extent to which consortship was based on male effort, and thus represented male mate-guarding^16^, we also noted for every minute who was the maintainer of the consortship. The maintainer was defined as the individual following the direction the consort partner is moving into. Whenever consort pairs were not moving, the last maintainer remained as maintainer until the next movement occurred.

### Faecal sample collection and hormone analysis

In parallel to behavioural observations, and for analysis of our behavioural data, we collected faecal samples for hormonal analysis opportunistically during the 31 ovarian cycles of the 19 focal females. Sample collection occurred on a daily basis at least during mid-cycle, i.e. the period of maximum anogenital swelling, and the seven following days, to allow ovulation to be timed accurately in each cycle^67^. Only samples uncontaminated by urine were collected directly after the animal had been observed defecating. Samples were placed on ice, and stored frozen (− 18 °C) within 10 h of collection. Overall, 3–7 samples per week were collected per female. From these samples we determined the timing of ovulation based on patterns of immunoreactive faecal progestogen concentrations measured by enzyme immunoassay (EIA). Processing of the samples and their EIA measurement has been described by^41^. Sensitivity of the assay at 90% binding was 20 pg/well. Inter-assay coefficients of variation (CVs) of high and low value quality controls were 15.4% (high) and 15.7% (low), while intra-assay CVs were 7.8% (high) and 9.5% (low). The presumed time of ovulation was determined by counting back from the defined post-ovulatory rise in faecal progestogen concentrations^68^, taking into account the excretion lag time of faecal progestogens^69^. In this way, a two day ovulation window was determined for all 31 cycles as the 2 and 3 days prior to the post-ovulatory rise in progestogens. In all 31 ovarian cycles, this rise was defined as a rise of more than 2 standard deviations above a baseline calculated from the previous 3–5 values, and which was sustained for a minimum of 3 datapoints (as in, e.g.^69^). We defined a four day fertile period as the 2 day ovulation window plus the previous 2 days (as in, e.g.^69^), and took the previous 5 days (pre-fertile phase) and the following 5 days (post-fertile phase) for comparison.

### Genetic analysis

We collected fecal samples non-invasively (up to 3 samples per individual) of offspring conceived during the study period 2006 and 2007 (N = 14), their mothers (N = 14) and all potential sires (N = 53, total 81 subjects) applying the two-step alcohol-silica storage protocol^70^. Potential sires were defined as any adult males present or immigrating into our study groups during our study period. We extracted DNA using 100–150 mg of faeces and the GENIAL all-tissue DNA extraction kit following the manufacturer’s instructions with only slight modification. Samples were genotyped on a total of twelve highly variable microsatellite markers (details in^71^). We used a combination of the multiple tube approach^72,73^ and the two-step multiplex PCR to increase the accuracy of the results^74^. Products were analyzed with an ABI PRISM3100 automated sequencer and the ABI peak scanner software. A heterozygous genotype was accepted when both alleles were confirmed at least two times per extract, i.e., a total of four independent PCRs were required for a given individual. A homozygous genotype was assigned when a single allele occurred in six independent PCRs, in order to control for allelic dropout^30,72,73^. In case one heterozygous genotype appeared within the six PCRs, we did up to eleven PCR replications to ensure that we reported a true genotype^72^.

Maternity derived from field observations was genetically tested and confirmed for all mother-infant pairs (N = 14) in our study which were subsequently used in the paternity analysis. For paternity assignment, we considered all genotyped males as potential sires for all infants (compare^71^) and compared an average of 11.36 ± 0.63 markers over the 14 mother-father-offspring trios (Table 1). We used a combination of exclusion and likelihood analyses (compare^71^) as follows: for 10 infants, all potential males were excluded on at least two loci, with the exception of the assigned sire, who matched the mother–offspring pair at all loci. In 4 cases, the assigned sire had one mismatch with the given infant, while the next likely sires had at least two mismatches. All offspring, paternity assignments were supported at the 95% confidence level by the likelihood method calculated by CERVUS 3.0^75^ (Table 1).

### Data analysis

*Aim 1: Female ovarian cycle synchrony:* Using our observations of sexual swellings, we assessed for each group separately, then averaged across the groups, overlap in: females exhibiting any swelling; females exhibiting maximal swelling; females being in the fertile phase of their conceptive cycle as hormonally assessed. The sample size was n = 31 cycles.

*Aims 2 and 3: Consortships and the maintenance of consortships:* To investigate the frequency with which we counted females and males as being in consort, and for analysis of the maintenance of consorts, we carried out Generalized Linear Mixed Models (GLMMs). Dependent count data were zero-inflated, as assessed by comparison of the number of zeros found in each variable compared with the number predicted under the Poisson distribution. This is because behaviours were absent on many days of the cycle. We therefore undertook zero-inflated Poisson models using the R package MCMCglmm^76^. Response variables were: Consort Count, or Male Maintenance Count or Mating Count, with an offset for number of observation counts to account for any differences in observation time. Fixed effects were cycle phase, and cycle type, with Female ID and Group specified as random effects. The models produce two different B values and p values for each variable—one for the Poisson part of the model and the other for the zero-inflated part which models the distribution of the excess zeros. Models were checked for convergence by checking trace plots. Models were carried out for all males and alpha-males separately, except for mating, for which only alpha-males are considered since analyses of how mating from all males relates to ovulatory timing has been already published^41^. When creating mean values for presentation, values were averaged within and then across cycles so that no one cycle exerted undue influence over the presented figures. The sample size was 314 female days from 31 cycles.

*Aim 4: Number of mating partners across the cycle:* To test whether females had more partners according to ‘cycle phase’ (pre-fertile vs fertile vs post-fertile phases), we carried out repeated-measures ANOVA comparing the number of partners obtained in each cycle for each phase, while also testing for differences between cycles by including ‘cycle type’ (conceptive vs non-conceptive) in the model. Following this, we carried out post-hoc paired t tests to test each cycle phase against the other. The sample size was n = 31 cycles.

*Aim 5: Mating skew*: To test for mating and consort skew by dominance rank we carried out Spearman’s correlations. As values for rank number 3 onwards were all close to zero or zero (see “Results”) we used only data for the first 6 ranks (the minimum number of data points required to undertake a Spearman’s correlation) in these analyses.

In order to calculate the predicted mating skew for males of different ranks based on the PoA model, we assessed female fertile period synchrony across all females in the group, with fertile phase determined either by hormonal data where available, or otherwise assessed as the days -5 to -2 prior to the onset of detumescence (day 0; Engelhardt et al. in prep.). On the basis of this, males were assigned a rank-specific probability of mating with a female on each day any female was fertile, where the alpha obtained a probability of 1 and all other males 0 when only one female was fertile, the alpha and beta males probabilities of 0.5 each and all other males 0 when two females were fertile, and so on. These values were then averaged over the observation period to obtain an estimate of the proportion of matings that males of each rank should obtain according to the PoA model. This estimate was then compared to the observed mating data. The sample size was n = 31 cycles.

*Aim 6: Reproductive skew*: To compare the reproductive skew of our study population with that of other species, we calculated Nonacs’ reproductive skew index (B-index^77^) and tested it for deviation from random distribution using the Skew calculator 2013 (https://www.eeb.ucla.edu/Faculty/Nonacs/PI.html). We also calculated the mean proportion of infants that were sired by the two alpha-males as this is a typical measure of reproductive skew in studies on primates (e.g.^10^). The sample size was n = 31 cycles.

## References

[1] Darwin C (1871). The Descent of Man and the Selection in Relation to Sex.

[2] Miller EJ, Eldridge MDB, Cooper DW, Herbert CA (2010). Dominance, body size and internal relatedness influence male reproductive success in eastern grey kangaroos (*Macropus giganteus*). Reprod. Fertil. Dev..

[3] Hirsch BT, Maldonado JE (2011). Familiarity breeds progeny: Sociality increases reproductive success in adult male ring-tailed coatis (*Nasua nasua*). Mol. Ecol..

[4] Natoli E, Schmid M, Say L, Pontier D (2007). Male reproductive success in a social group of urban feral cats (*Felis catus* L.). Ethology.

[5] Clutton-Brock T, Isvaran K (2006). Paternity loss in contrasting mammalian societies. Biol. Lett..

[6] Altmann SA (1962). A field study of the sociobiology of rhesus monkeys, *Macaca mulatta*. Ann. N. Y. Acad. Sci..

[7] Kutsukake N, Nunn CL (2006). Comparative tests of reproductive skew in male primates: The roles of demographic factors and incomplete control. Behav. Ecol. Sociobiol..

[8] Ostner J, Nunn CL, Schülke O (2008). Female reproductive synchrony predicts skewed paternity across primates. Behav Ecol.

[9] Janson C, Verdolin J, Brockmann DK, Van Schaik C (2005). Seasonality of primate births in relation to climate. Seasonality in Primates—Studies of Living and Extinct Human and Non-human Primates.

[10] Gogarten JF, Koenig A (2012). Reproductive seasonality is a poor predictor of receptive synchrony and male reproductive skew among nonhuman primates. Behav. Ecol. Sociobiol..

[11] Brockmann DK, Van Schaik CP, Brockmann DK, Van Schaik CP (2005). Seasonality and reproductive function. Seasonality in Primates: Studies of Living and Extinct Human and Non-human Primates.

[12] Sterck EHM, Watts DP, van Schaik CP (1997). The evolution of female social relationships in nonhuman primates. Behav. Ecol. Sociobiol..

[13] Nunn CL (1999). The number of males in primate social groups: A comparative test of the socioecological model. Behav. Ecol. Sociobiol..

[14] Carnes LM, Nunn CL, Lewis RJ (2011). Effects of the distribution of female primates on the number of males. PLoS One.

[15] Manson JH (1997). Primate consortships: A critical review. Curr. Anthropol..

[16] Andersson MB (1994). Sexual Selection.

[17] Fürtbauer I, Heistermann M, Schülke O, Ostner J (2011). Concealed fertility and extended female sexuality in a non-human primate (*Macaca assamensis*). PLoS One.

[18] Plavcan JM (2011). Understanding dimorphism as a function of changes in male and female traits. Evol. Anthropol. Issues News Rev..

[19] Setchell JM, Charpentier M, Wickings EJ (2005). Mate guarding and paternity in mandrills: Factors influencing alpha male monopoly. Anim. Behav..

[20] Bradley BJ (2005). Mountain gorilla tug-of-war: Silverbacks have limited control over reproduction in multimale groups. Proc. Natl. Acad. Sci. USA.

[21] Nunn CL (1999). The evolution of exaggerated sexual swellings in primates and the graded-signal hypothesis. Anim. Behav..

[22] Rodriguez-Llanes JM, Verbeke G, Finlayson C (2009). Reproductive benefits of high social status in male macaques (Macaca). Anim. Behav..

[23] Paul A, Kuester J, Timme A, Arnemann J (1993). The association between rank, mating effort and reproductive success in male Barbary macaques (*Macaca sylvanus*). Primates.

[24] Kümmerli R, Martin RD (2005). Male and female reproductive success in *Macaca sylvanus* in Gibraltar: No evidence for rank dependence. Int. J. Primatol..

[25] Brauch K (2008). Sex-specific reproductive behaviours and paternity in free-ranging Barbary macaques (*Macaca sylvanus*). Behav. Ecol. Sociobiol..

[26] Berard JD, Nurnberg P, Epplen JT, Schmidtke J (1994). Alternative reproductive tactics and reproductive success in male rhesus macaques. Behaviour.

[27] Widdig A (2004). A longitudinal analysis of reproductive skew in male rhesus macaques. Proc. Biol. Sci..

[28] Dubuc C, Muniz L, Heistermann M, Engelhardt A, Widdig A (2011). Testing the priority-of-access model in a seasonally breeding primate species. Behav. Ecol. Sociobiol..

[29] de Ruiter JR, van Hooff JARAM, Scheffrahn W (1994). Social and genetic aspects of paternity in wild long-tailed macaques (*Macaca fascicularis*). Behaviour.

[30] Engelhardt A, Heistermann M, Hodges JK, Nuernberg P, Niemitz C (2006). Determinants of male reproductive success in wild long-tailed macaques (*Macaca fascicularis*)—male monopolisation, female mate choice or post-copulatory mechanisms?. Behav. Ecol. Sociobiol..

[31] Plavcan JM, van Schaik CP (1997). Intrasexual competition and body weight dimorphism in anthropoid primates. Am. J. Phys. Anthropol..

[32] Plavcan JM, van Schaik CP, Kappeler PM (1995). Competition, coalitions and canine size in primates. J. Hum. Evol..

[33] Groves C (2001). Primate Taxonomy.

[34] Thierry B, Iwaniuk AN, Pellis SM (2000). The influence of phylogeny on the social behaviour of macaques (Primates: Cercopithecidae, genus Macaca). Ethology.

[35] Duboscq J (2013). Social tolerance in wild female crested macaques (*Macaca nigra*) in Tangkoko-Batuangus Nature Reserve, Sulawesi, Indonesia. Am. J. Primatol..

[36] Plavcan JM, van Schaik CP, McGraw WS, van Schaik CP, Brockman DK (2005). Seasonality, social organization, and sexual dimorphism in primates. Seasonality in Primates: Studies of Living and Extinct Human and Non-Human Primates.

[37] Marty PR, Hodges K, Agil M, Engelhardt A (2017). Alpha male replacements and delayed dispersal in crested macaques (*Macaca nigra*). Am. J. Primatol..

[38] Kerhoas D, Perwitasari-Farajallah D, Agil M, Widdig A, Engelhardt A (2014). Social and ecological factors influencing offspring survival in wild macaques. Behav. Ecol..

[39] Neumann C, Assahad G, Hammerschmidt K, Perwitasari-Farajallah D, Engelhardt A (2010). Loud calls in male crested macaques, *Macaca nigra*: A signal of dominance in a tolerant species. Anim. Behav..

[40] Martinez-Iñigoa L, Agil M, Engelhardt A, Pilot M, Majolo B (2017). Resource and mate defence influence the outcome of intergroup encounters in wild crested macaques (*Macaca nigra*). Primate Eye.

[41] Higham JP (2012). Sexual signalling in female crested macaques and the evolution of primate fertility signals. BMC Evol. Biol..

[42] Engelhardt A, Perwitasari-Farajallah D (2008). Reproductive biology of Sulawesi crested black macaques (*Macaca nigra*). Folia Primatol. (Basel).

[43] Marty PR, Hodges K, Agil M, Engelhardt A (2016). Determinants of immigration strategies in male crested macaques (*Macaca nigra*). Sci. Rep..

[44] Wigby S, Chapman T (2004). Sperm competition. Curr. Biol..

[45] Tregenza T, Wedell N (1998). Benefits of multiple mates in the cricket gryllus bimaculatus. Evolution.

[46] Clutton-Brock TH (1998). Reproductive skew, concessions and limited control. Trends Ecol. Evol..

[47] Alberts SC, Buchan JC, Altmann J (2006). Sexual selection in wild baboons: From mating opportunities to paternity success. Anim. Behav..

[48] Boesch C, Kohou G, Néné H, Vigilant L (2006). Male competition and paternity in wild chimpanzees of the Taï forest. Am. J. Phys. Anthropol..

[49] Higham JP, Heistermann M, Maestripieri D (2011). The energetics of male-male endurance rivalry in free-ranging rhesus macaques, *Macaca mulatta*. Anim. Behav..

[50] Muniz L (2010). Male dominance and reproductive success in wild white-faced capuchins (*Cebus capucinus*) at Lomas Barbudal, Costa Rica. Am. J. Primatol..

[51] Strier KB, Chaves PB, Mendes SL, Fagundes V, Di Fiore A (2011). Low paternity skew and the influence of maternal kin in an egalitarian, patrilocal primate. Proc. Natl. Acad. Sci..

[52] Daspre A, Heistermann M, Hodges JK, Lee PC, Rosetta L (2009). Signals of female reproductive quality and fertility in colony-living baboons (*Papio hanubis*) in relation to ensuring paternal investment. Am. J. Primatol..

[53] Weingrill T, Lycett JE, Barrett L, Hill RA, Henzi SP (2003). Male consortship behaviour in chacma baboons: The role of demographic factors and female conceptive probabilities. Behaviour.

[54] Engelhardt A (2004). Assessment of female reproductive status by male longtailed macaques, *Macaca fascicularis*, under natural conditions. Anim. Behav..

[55] Higham JP, Semple S, MacLarnon A, Heistermann M, Ross C (2009). Female reproductive signaling, and male mating behavior, in the olive baboon. Horm. Behav..

[56] Schülke O, Ostner J (2008). Male reproductive skew, paternal relatedness, and female social relationships. Am. J. Primatol..

[57] Schülke O, Ostner J, Mitani JC, Call J, Kappeler PM, Palombit RA, Silk JB (2012). Ecological and social influences on sociality. The evolution of Primate Societies.

[58] Higham, J. P. *et al.* Female fertile phase synchrony, and male mating and reproductive skew, in the crested macaque. Dryad, Dataset. 10.5061/dryad.rfj6q578x. (2021). 10.1038/s41598-021-81163-1 PMC7896048 33608592

[59] Rosenbaum B, O’Brien TG, Kinnaird M, Supriatna J (1998). Population densities of Sulawesi crested black macaques (*Macaca nigra*) on Bacan and Sulawesi, Indonesia: Effects of habitat disturbance and hunting. Am. J. Primatol..

[60] Collins NM (1991). The Conservation Atlas of Tropical Forests: Asia and the Pacifics.

[61] O’Brien TG, Kinnaird MF (1997). Behavior, diet, and movements of the Sulawesi crested black macaque (*Macaca nigra*). Int. J. Primatol..

[62] Kinnaird MF, O’Brien TG (1999). A contextual analysis of the loud call of the Sulawesi crested black macaque, *Macaca nigra*. Trop. Biodivers..

[63] Neumann C (2011). Assessing dominance hierarchies: Validation and advantages of progressive evaluation with Elo-rating. Anim. Behav..

[64] Hadidian J, Bernstein IS (1979). Female reproductive cycles and birth data from an Old World monkey colony. Primates.

[65] Altmann J (1974). Observational study of behavior: Sampling methods. Behaviour.

[66] Danish LM, Palombit RA (2014). “Following”, an alternative mating strategy used by male olive baboons (*Papio hamadryas anubis*): Quantitative behavioral and functional description. Int. J. Primatol..

[67] Hodges JK, Heistermann M (2011). Field Endocrinology: Monitoring Hormonal Changes in Free-Ranging Primates.

[68] Heistermann M (2001). Loss of oestrus, concealed ovulation and paternity confusion in free-ranging Hanuman langurs. Proc. Biol. Sci..

[69] Engelhardt A, Hodges JK, Niemitz C, Heistermann M (2005). Female sexual behavior, but not sex skin swelling, reliably indicates the timing of the fertile phase in wild long-tailed macaques (*Macaca fascicularis*). Horm. Behav..

[70] Nsubuga AM (2004). Factors affecting the amount of genomic DNA extracted from ape faeces and the identification of an improved sample storage method. Mol. Ecol..

[71] Engelhardt A, Muniz L, Perwitasari-Farajallah D, Widdig A (2017). Highly polymorphic microsatellite markers for the assessment of male reproductive skew and genetic variation in Critically Endangered crested macaques (*Macaca nigra*). Int. J. Primatol..

[72] Taberlet P (1996). Reliable genotyping of samples with very low DNA quantities using PCR. Nucleic Acids Res..

[73] Taberlet P, Luikart G (1999). Non-invasive genetic sampling and individual identification. Biol. J. Linn. Soc..

[74] Arandjelovic M (2009). Two-step multiplex polymerase chain reaction improves the speed and accuracy of genotyping using DNA from noninvasive and museum samples. Mol. Ecol. Resour..

[75] Kalinowski ST, Taper ML, Marshall TC (2007). Revising how the computer program CERVUS accommodates genotyping error increases success in paternity assignment. Mol. Ecol..

[76] Hadfield JD (2010). MCMC methods for multi-response generalized linear mixed models: The MCMCglmm package. J. Stat. Softw..

[77] Nonacs P (2003). Measuring the reliability of skew indices: Is there one best index?. Anim. Behav..

